# Specific Tandem 3'UTR Patterns and Gene Expression Profiles in Mouse Thy1^+^ Germline Stem Cells

**DOI:** 10.1371/journal.pone.0145417

**Published:** 2015-12-29

**Authors:** Yan Huang, Yuanyan Xiong, Zhuoheng Lin, Xuyang Feng, Xue Jiang, Zhou Songyang, Junjiu Huang

**Affiliations:** 1 Key Laboratory of Reproductive Medicine of Guangdong Province, the First Affiliated Hospital and Key Laboratory of Gene Engineering of the Ministry of Education, School of Life Sciences, Sun Yat-sen University, Guangzhou, 51000, China; 2 SYSU-CMU Shunde International Joint Research Institute, Shunde, China; Rutgers New Jersey Medical School, UNITED STATES

## Abstract

A recently developed strategy of sequencing alternative polyadenylation (APA) sites (SAPAS) with second-generation sequencing technology can be used to explore complete genome-wide patterns of tandem APA sites and global gene expression profiles. spermatogonial stem cells (SSCs) maintain long-term reproductive abilities in male mammals. The detailed mechanisms by which SSCs self-renew and generate mature spermatozoa are not clear. To understand the specific alternative polyadenylation pattern and global gene expression profile of male germline stem cells (GSCs, mainly referred to SSCs here), we isolated and purified mouse Thy1^+^ cells from testis by magnetic-activated cell sorting (MACS) and then used the SAPAS method for analysis, using pluripotent embryonic stem cells (ESCs) and differentiated mouse embryonic fibroblast cells (MEFs) as controls. As a result, we obtained 99,944 poly(A) sites, approximately 40% of which were newly detected in our experiments. These poly(A) sites originated from three mouse cell types and covered 17,499 genes, including 831 long non-coding RNA (lncRNA) genes. We observed that GSCs tend to have shorter 3'UTR lengths while MEFs tend towards longer 3'UTR lengths. We also identified 1337 genes that were highly expressed in GSCs, and these genes were highly consistent with the functional characteristics of GSCs. Our detailed bioinformatics analysis identified APA site-switching events at 3'UTRs and many new specifically expressed genes in GSCs, which we experimentally confirmed. Furthermore, qRT-PCR was performed to validate several events of the 334 genes with distal-to-proximal poly(A) switch in GSCs. Consistently APA reporter assay confirmed the total 3'UTR shortening in GSCs compared to MEFs. We also analyzed the cis elements around the proximal poly(A) site preferentially used in GSCs and found C-rich elements may contribute to this regulation. Overall, our results identified the expression level and polyadenylation site profiles and these data provide new insights into the processes potentially involved in the GSC life cycle and spermatogenesis.

## Introduction

Alternative splicing of pre-mRNAs can generate multiple mRNA isoforms from a single gene, and it has been shown that more than half of human and mouse genes use alternative polyadenylation (APA) sites to produce multiple isoforms with different poly(A) sites [1, 2]. Studies in other model species have also shown that the majority of genes undergo APA events in the process of producing alternative mRNAs, including yeast, Drosophila, zebrafish, and *C*. *elegans* et al [3–5]. The poly(A) sites include post-transcriptional regulatory elements in the mature transcript that may significantly change mRNA stability, localization, or translation, thereby impacting the protein sequences and the amount of protein derived from that mRNA.

Microarrays and deep sequencing of poly(A) tail methods, such as PAS-Seq, 3P-seq [3], SAPAS [6] and 3'READS [7], have recently been used to identify poly(A)s on a genome-wide scale. These methods have been applied to compare systematic changes in mRNA 3'end length under multiple conditions, such as during proliferation, differentiation, development, disease and in response to extracellular changes. It has been shown that proliferating T lymphocytes, neurons and tumor cells globally use shorter 3'UTRs, and this is likely due to the benefit gained from the increased stability conferred by UTR-based mRNA regulation processes, such as the loss of microRNA binding sites [8–10].

Different tissues have distinct APA usage profiles. For example, the genes in the brain have longer 3'UTRs, while those in testes have shorter 3'UTRs [11–13]. Male mammals can maintain their reproductive abilities throughout most of their life because they have spermatogonial stem cells (SSCs). Originating from primordial germ cells (PGCs), SSCs are the only adult stem cells that transmit genetic information to subsequent generations. During spermatogenesis, SSCs differentiate into mature spermatozoa, which can fertilize an oocyte from a female to form a zygote. Embryonic stem cell (ESC) lines are derived from blastocysts, while induced pluripotent stem cells (iPSCs) are derived from reprogramming and resemble ESCs in their pluripotency and gene expression patterns. Moreover, during reprogramming from mouse embryonic fibroblasts (MEFs) to iPSCs, it was shown that the 3'UTR became shorter, resembling their state in ESCs [14]. Thus, 3'UTR length may also correlate with differentiation state. However, the gene expression profile of GSCs remains unknown. Thus, we decided to examine the differential APA usage patterns and expression profiles among GSCs, ESCs and differentiating MEFs.

In this study, we used SAPAS to conduct a genome-wide analysis of changes in 3'-end profiles, including 3'UTR length and expression levels for 16,668 mRNAs and 831 lncRNA genes in GSCs, ESCs and MEFs. Through a comparative analysis of gene expression in three samples, genes detected as highly expressed in GSCs were analyzed for gene ontology (GO) annotation, and the results were significantly consistent with the functional characteristics of GSCs. In addition, we found that many APA events correlate with GSCs, and an overall 3'UTR shortening occurs in GSCs. Cis elements enriched upstream of alternatively used poly(A) sites may contribute to the regulation of coordinated transcript shortening events in GSCs. By using SAPAS, we provide the first global and quantitative analysis of polyA site profiles specifically in GSCs.

## Materials and Methods

### Cells Collection

All animal procedures were approved by the Institutional Animal Care and Use Committees of Sun Yat-sen University. Primary mouse embryonic fibroblasts (MEFs) were isolated from 12.5 days postcoitum (dpc) C57 fetuses and cultured for 2 passages *in vitro* before collection. Mouse ESC line N33 was derived from C57 mice as described previously [15]. The pluripotency of N33 ESCs has been tested through the production of ESC mice. For male GSC isolation, we followed Brinster’s protocol, which includes magnetic-activated cell sorting (MACS) to isolate Thy1^+^ GSCs from 3-week- old Oct4-EGFP mice and wild type C57 mice. Briefly, mouse testes were removed from the tunica albuginea and then individually digested with collagenase IV (Invitrogen, 17104–019) and 0.25% Trypsin-EDTA (Gibco) plus DNase I (Sigma, DN25). After filtering with 70μm and 40μm filters, single cell suspensions were centrifuged in 30% Percoll to collect GSC-enriched cell pellets for MACS. MEFs, ESCs and Thy1^+^ positive GSCs were transferred to TRIzol for RNA extraction. For RT-PCR validation, mouse spleens were digested and Thy1^+^ cells were isolated for negative controls.

### Poly(A)-Seq library construction and sequencing

A sequencing library was constructed as described previously [6]. Briefly, total RNA was extracted from cells by TRIzol. Approximately ~10μg of total RNA was randomly fragmented by heating. An anchored oligo d(T) primer and a 5' template-switching linker tagged with Illumina adaptors were used for template-switch reverse transcription (RT) by SuperScript II reverse transcriptase from Invitrogen. PCR was then performed to amplify the cDNA and to introduce two mutations into the poly(A). By using a determined number of cycles, we ensured that the ds cDNA remained in the exponential phase of amplification. The PCR products were recovered after PAGE, and the size-selection for 250–400 bp fragments was performed by PAGE gel excision. The final pooled fragments were sequenced from the 3'end with an Illumina GA IIx.

### Data analysis

#### 1. Read mapping, internal priming filtering and poly(A) site clustering

Raw sequenced reads were trimmed of linkers and preceding Ts, and reads with a quality score<15 were defined as low quality reads and removed. This left clean reads, which were aligned to the mouse genome (mm9) and splice junctions (generated from UCSC gene annotation) using Bowtie (version2). Uniquely aligned reads with no more than one mismatch were retained. Then, reads that had more than 12 As in the 20 nt immediately closest to the5'-end of the poly(A) junction (cleavage site), which were considered possible internal priming artifacts, were discarded. The remaining uniquely mapped reads, which were named as used reads, were used for further analysis. To identify poly(A) sites, the 5' ends of the used reads from 3 cell lines were pooled and clustered if they were located within 40nt of each other. We used RPM (reads per million), calculated as the number of reads assigned to a position per million used reads in the sample, to estimate the expression level of each cluster. When the cluster size was >40 nt, we split the cluster according to the highest RPM supported site until all of the used reads were clustered. The cluster with the highest supported RPM in 3 samples was defined as the poly(A) site for the poly(A) cluster, and a minimum of 5 RPM in a cluster was required.

#### 2. Annotation of the identified poly(A) sites

Genome annotation of the identified poly(A) sites was carried out using mouse genomic sequences and gene loci information based on UCSC exon locus annotation (http://hgdownload.cse.ucsc.edu/goldenPath/mm9/database/) and Ensembl gene annotations (BioMart, http://www.biomart.org/, Release 72). The annotation order was 3'UTR, Exon, Intron, 5'UTR, downstream (10 kb from the 3'UTR end), non-coding genes, mitochondrial regions, antisense regions and intergenic regions. Poly(A) sites in mitochondrial regions were not used for further analysis. The remaining poly(A) sites were defined as known poly(A) sites, EST poly(A) sites and novel poly(A) sites according to their overlap (or absence of overlap) with annotated poly(A) sites (within 30 bp of the 3’UTR end) or Mouse PolyA_DB sites (poly(A) sites detected by EST data in previously published literature [16]).

#### 3. Gene expression and 3'UTR pattern comparison

Each gene boundary was obtained according to gene annotations to determine the largest potential genomic region, and each gene region was extended to 3000 bp at most without causing overlap with another. Gene expression levels were calculated as the sum of all of the poly(A) cluster expression within the gene boundary. The genes with significant P-values corresponding to a false discovery rate cutoff of <0.01 and RPM fold change >2 were chosen as significantly differentially expressed genes. Fisher’s exact test was used to compute the probability of differentially expressed genes' read counts between samples, and the Benjamini-Hochberg false discovery rate (FDR) was estimated.

To compare the different APA patterns in unique 3'UTRs between samples, we first extended the 3'UTR region to 3000 bp without causing overlap with other genes. We then grouped 3'UTRs by their stop codons. The 3'UTRs that shared the same stop codons were grouped, but the overlapped 3'UTRs were discarded. For mRNA genes with more than one poly(A) site in the unique 3'UTR region, the two most abundant isoforms were used for APA analysis. For lncRNAs, the two most abundant isoforms within the gene boundary were used. Fisher’s exact test with a Bonferroni correction was used, as described above, to examine whether the abundance of two isoforms was significantly different between two cell lines. The genes with a corrected p-value of <0.01 and an absolute level change >5% were determined to be significantly different alternative poly(A) site usages. All statistical analyses were performed in R.

The functional annotation of differentially expressed or differentially APA-regulated genes in the KEGG pathway and via Gene Ontology (biological processes) was performed using DAVID bioinformatics resources. Probabilities were evaluated by Bonferroni correction, and values less than 0.01 were considered significant and the top 20 were listed in the supplemental figures.

#### 4. PAS motif and cis elements analysis

Regions around the polyadenylation sites (PAS) (±100 nt) were scanned for 6-mer motifs, and significant hexamer frequencies were identified by comparing them to those expected by chance from those in all regions by nucleotide composition. Significant hexamers were collected iteratively. Once the most significant 6-mer was identified, all fragments containing this motif were removed and those remaining were used to seek the next most frequent hexamer, until the top ten most prominent PAS motifs were identified. For the cis elements analysis, the genes with a preferential proximal poly(A) site in GSCs compared to MEFs and ESCs were selected. The -500nt to +500nt regions surrounding the poly(A) site were divided equally into five subregions. Motifs were analyzed using Multiple Em for Motif Elicitation (MEME) Software. E values representing the statistical significance of the motif were ranked in order. The most statisticallyf significant (low E-value) motifs were shown first.

### Raw data download

The raw poly(A)-Seq sequence data can be download from http://songyanglab.sysu.edu.cn/download/ssc.

### RT-PCR validation of gene expression

Briefly, the total RNAs of tissues or cells were isolated with the TRIzol (Invitrogen) and purified with an RNeasy mini Kit (QIAGENE), reverse-transcribed into cDNA using an iScript cDNA Synthesis Kit (Bio-Rad), and amplified using an ABI PCR system (Applied Biosystems).

### qRT-PCR validation of poly(A) switched genes and APA reporter analysis

For qRT-PCR validation of poly(A) switched genes, two most expressed poly(A) sites were selected as proximal /distal sites according to the locus. F1/R1 Primers are designed within 1000bp upstream of the proximal sites while F2/R2 Primers are designed between the two poly(A) sites (arrows). For each gene, Real-time PCR were performed using two pairs of primers F2R2 and F1R1, and the relative signal for primer set F2R2/F1R1 ratios was calculated. For APA reporter analysis, reporters 77S.AE and 77S.AD were constructed previously derived from human CSTF3 and contains UGUA, AUUAAA, and U-rich elements. 77S.AE had additional UGUG element as a stronger poly(A) site than 77S.AD [14]. Two reporter constructs were transfected separately into cultured GSCs and MEFs by ViaFect reagent (Promega) according to the manual protocol. Fourty-eight hours later qRT-PCR was performed with primers targeting either the proximal (RFP) or distal (EGFP) regions of APA site, the relative expression levels of two transcript variants resulting from APA were calculated.

## Results

### Poly(A) site profiles in mouse GSCs, ESCs and MEFs

To analyze the polyadenylation pattern and the expression profile of mouse GSCs compared to other cell types, we use the SAPAS method to obtain high-resolution poly(A) site information and gene expression profiles in GSCs, ESCs and MEFs.

**Fig 1:**
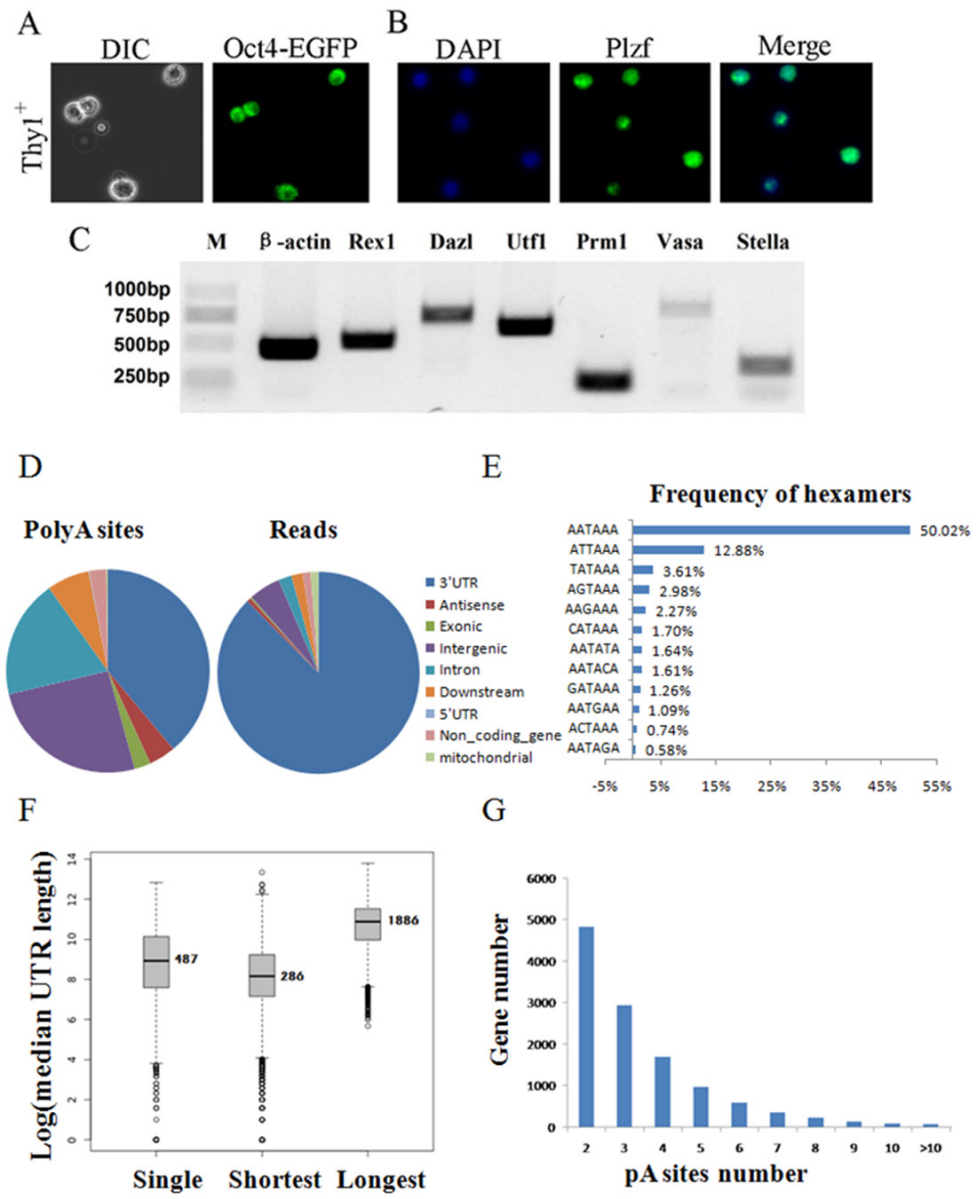
Sample preparation and annotation of SAPAS data. (A) MACS-isolated Thy1+ male germline stem cells (GSCs) from Oct4-EGFP male mice were GFP positive. (B) MACS-isolated wildtype C57 GSCs are PLZF positive, as shown by immunostaining. (C) RT-PCR showed the expression of the internal control (β-actin) and PGC-specific genes in the GSC sample. (D) Pie charts showing the distribution of poly(A) sites and poly(A) reads in the genome. (E) Frequencies of poly(A) sites containing the PAS motif AAUAAA or other top hexamers in the 10 to 40 nt region upstream of the poly(A) sites. (F) Histograms of the distances between stop codons and poly(A) (median = 487) sites in genes with single poly(A) sites (6657 genes) and distances between stop codons and closest poly(A) sites (median = 286) and longest poly(A) sites (median = 1886) in genes with APA in the 3'-end (8611 genes). (G) Bar graph showing the poly(A) site distribution of genes in 3 cell lines.

To isolate GSCs from mice, we designed a MACS method using a Thy1^+^ antibody. To confirm that Thy1^+^ cells included GSCs, we first isolated Thy1^+^ cells from Oct4-EGFP mice, which express Enhanced Green Fluorescent Protein (EGFP) under the control of the Oct4 promoter and distal enhancer. Oct4 is a well-known key marker of adult stem cells. We found that most of the Thy1^+^ cells isolated from Oct4-EGFP mice testes were EGFP positive (Fig 1A), indicating that our method was successful. Then, we isolated approximately 4x10^6^ Thy1^+^ cells from wild type C57 mice. Before sending out the samples to generate a library, we used an antibody for the SSC marker Plzf to perform immunofluorescence (Fig 1B) and did RT-PCR to confirm the expression of some typical GSC markers, including Rex1, Dazl, Utf1, Prm1, Vasa and Stella (Fig 1C).

We next generated libraries of poly(A) tails from poly(A)^+^ RNAs from GSCs, ESCs and MEFs and subjected them to single-end deep sequencing. A total of >78.5 million (M) 3' transcript terminal reads were sequenced, after mapping them to the mouse genome (including junctions) and conducting internal priming filtering, ~12.7 M, ~11.8 M and ~5.6 M uniquely mapped reads were obtained for GSCs, MEFs and ESCs, respectively (Table 1). Matched sequences from the 3 samples were pooled to identify poly(A) sites. As described in the methods, reads within 40 nt were clustered as candidate poly(A) sites. For each library, we normalized the number of reads aligned to the poly(A) clusters to uniquely aligned read numbers in the library and measured the normalized value as a poly(A) site expression level called reads per million (RPM). The clusters that had a total RPM > 0.5 in 3 libraries remained, and the most frequently implicated sites were identified as poly(A) sites. As a result, we obtained 99,944 poly(A) sites. We next compared poly(A) sites detected by our method with those detected via the PAS-Seq method for mouse cell lines (ESCs, neural stem/progenitor (NSPs), and neurons) [10]. We found that over 50% of the poly(A) sites were within 40 nt of each other, and ~ 40% of the poly(A) sites were newly detected in our study. As expected, of the total poly(A) sites, ~30% were mapped to the annotated 3'UTR or the 10K downstream region of annotated genes (~25% mapped to the 3'UTR region), and these poly(A) clusters represented ~80% of all of the uniquely mapped reads. As show in Fig 1D, the rest of the poly(A) sites were mapped to exons (2.5%), introns (18.9%), intergenic regions (25.7%), antisense transcripts (4.2%) and non-coding RNA genes (2.6%), which accounted for 9.3% of mapped reads. Overall, coding regions and 5'UTRs contained the fewest poly(A) sites, and less than 5.1% of reads mapped to un-annotated regions. Approximately 25% of the poly(A) sites were known poly(A) sites that accounted for ~55% of mapped reads. These results revealed a good match between our detected poly(A) sites and the known poly(A) sites that are supported by PAS-Seq data and reference poly(A) sites. Still, the polyadenylation signal hexamer AAUAAA and its close variants were identified in the region 10–40 nt upstream of most of the poly(A) sites (over 80%). The sequence composition (**Table A in** S1 File) and frequencies of these hexamers were very consistent with the findings reported by Tian [2] (Fig 1E).

**Table 1:** SAPAS data summery.

	MEFs	ESCs	GSCs
Raw reads	28712111	18439073	31369651
After polyNT control	26854098	16573707	29617141
Mapped to genome	22915995	12307629	22900002
Uniquely mapped to genome	11801336	5599789	12737851
Mapped to transcriptome (junction)	4857	4146	9161
Mapped to mitochondrial genome	137142	92111	181979
Mapped to nuclear genome	11806193	5603935	12747012
After internal primer filter	10445326	4996966	10528656
Cleavage clusters	70803	69983	80914
Known RefSeq poly(A) sites	17018	16411	18475
Genes sampled by cleavage clusters	15461	15312	16673
Total reads of poly(A) sites	1182635	779141	1252137

GSCs, germline stem cells; MEFs, mouse embryonic fibroblast cells; ESCs, embryonic stem cells.

The poly(A) sites identified from the three mouse cell types covered 17,499 genes, including 831 lncRNA genes. The poly(A)s in mRNA and lncRNA genes were supported by similar *cis* elements. We then grouped the annotated 3'UTRs by stop codon, as described in the methods, and combined these isoforms with gene data. The distribution of distances between poly(A) sites and stop codons had a peak at 884 nt and a median value of 947 nt, and the distances between the stop codon and the first (or proximal) and last (or distal) poly(A) sites had median values of 286 and 1886, respectively (Fig 1F). Of the 17,499 poly(A) sites covered by expressed genes, up to 8611 of the genes had more than one poly(A) site in the group 3'UTR, with 7986 of protein coding genes and 625 of the lncRNA genes undergoing alternative polyadenylation. On average, 2.6 poly(A) sites were detected per gene (Fig 1G).

### Comparison of poly(A) site usage profiles in mouse GSCs, ESCs and MEFs

**Fig 2:**
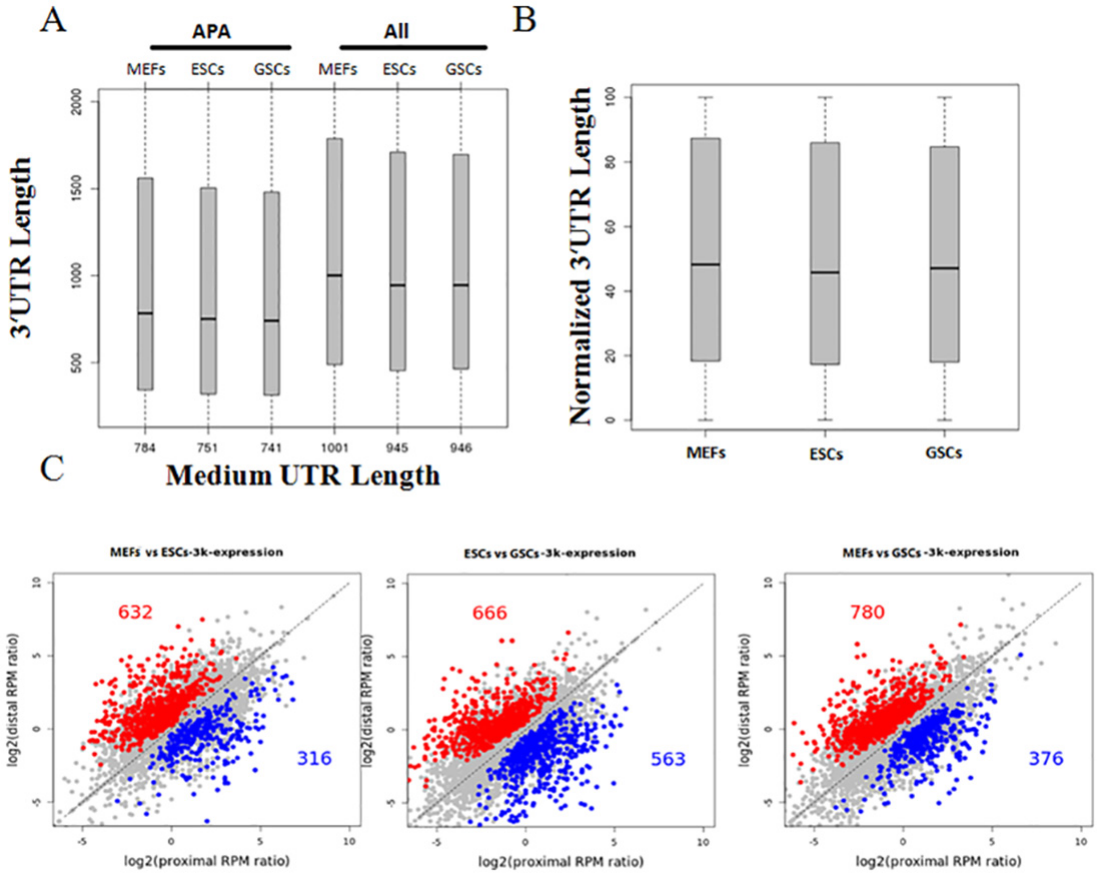
Analysis of alternative polyadenylation by SAPAS data. (A) Distribution of 3'UTR lengths of all mRNA genes and APA genes in the different cell lines. (B) Distribution of normalized 3'UTR lengths of genes with multi-poly(A) sites in the 3 samples. (C) Regulation of alternative pAs in the 3'-most exon of mRNA genes and in the whole gene of lncRNA genes.

We next used our 3'-end sequencing data to study the dynamic changes in overall 3'UTR lengths and the regulation of APA in GSCs, ESCs and MEFs. We defined the 3'UTR length of a gene as the weighted average length of all 3'UTRs in the 3'-most exon in the mRNA of the gene. As a result, MEFs were found to have longer 3'UTRs than ESCs, and GSCs had the shortest length of UTRs (Fig 2A). The 3'UTR lengthening bias was observed in MEFs when compared to ESCs and GSCs. To determine whether this pattern was due to alternative polyadenylation, we compared the 3'UTR length between cell lines only using the genes with more than one poly(A) isoform. Still, to reduce the effect of large values, the normalized 3'UTR average (normalized by the longest 3'UTR length) was used, and it showed the same average dynamic 3'UTR pattern (Fig 2B). As a result, we concluded that APA contributed to dynamic 3'UTR changes between cell lines.

We then focused on mRNA genes and lncRNA genes harboring APA isoforms for a systematic analysis of genes with significant 3'UTR length changes between cell lines. For mRNA genes, we performed pairwise comparisons between the most expressed two poly(A)s in the grouped unique 3'UTR region between cell lines. For the lncRNAs, the two most expressed poly(A)s in the whole gene region were compared. Statistically significant changes were identified using Fisher’s Exact Test and BH corrections, with a P value <0.01 and an abundance change > 5% considered to be significantly differentially regulated. Overall, the number of genes with relatively up-regulated distal poly(A) isoforms is significantly outnumbered (approximately 2-fold greater) by those having relatively up-regulated proximal poly(A) isoforms in differentiated MEFs compared to GSCs and ESCs (Fig 2C, left and right panel). In addition, similar numbers of up-regulated distal and proximal poly(A) isoforms in GSCs vs. ESCs were detected (Fig 2C, middle panel). Proximal-to-distal switching events were much more common than distal-to-proximal switch events in MEFs compared to the other two cell lines, suggesting that 3'UTRs were longer in MEFs. This result was consistent with previous reports that indicated the occurrence of 3'UTR lengthening during mouse cell differentiation and human stem cell differentiation and suggested that the testes tend to have shorter 3'UTRs than other tissues [7, 10, 14]. In addition, we used the statistical method developed previouly [17], in which all of the poly(A) sites in a 3'UTR group are considered, to identify APA switched genes between samples. A total of 502 genes were found to display differential poly(A) site switch in GSCs compared to ESCs or MEF. Consistently, we found 334 genes with distal-to-proximal 3'UTR switch (**Table B in** S1 File), which are much more than 168 genes with proximal-to-distal 3'UTR switch (**Table C in** S1 File).

To demonstrate the function of these significant APA site-switching events, we then performed GO enrichment analysis on 334 genes found to display up-regulated proximal poly(A) in GSCs by using the web accessible DAVID program. GO terms related to "cell cycle", "nuclear division", and "mitosis" were significantly enriched (**Fig A in** S2 File). There were 239 genes with 3'UTRs with distal-to-proximal switch events in both GSC vs. MEF and ESC vs. MEF comparisons, revealing the functional enrichment of genes involved in mitosis (**Fig B in** S2 File). These results suggest that APAs are tightly regulated in genes involved in the cell cycle and indicate an important regulatory role for APAs during differentiation in GSCs.

### Comparison of gene expression profiles in mouse GSCs, ESCs and MEFs

**Fig 3:**
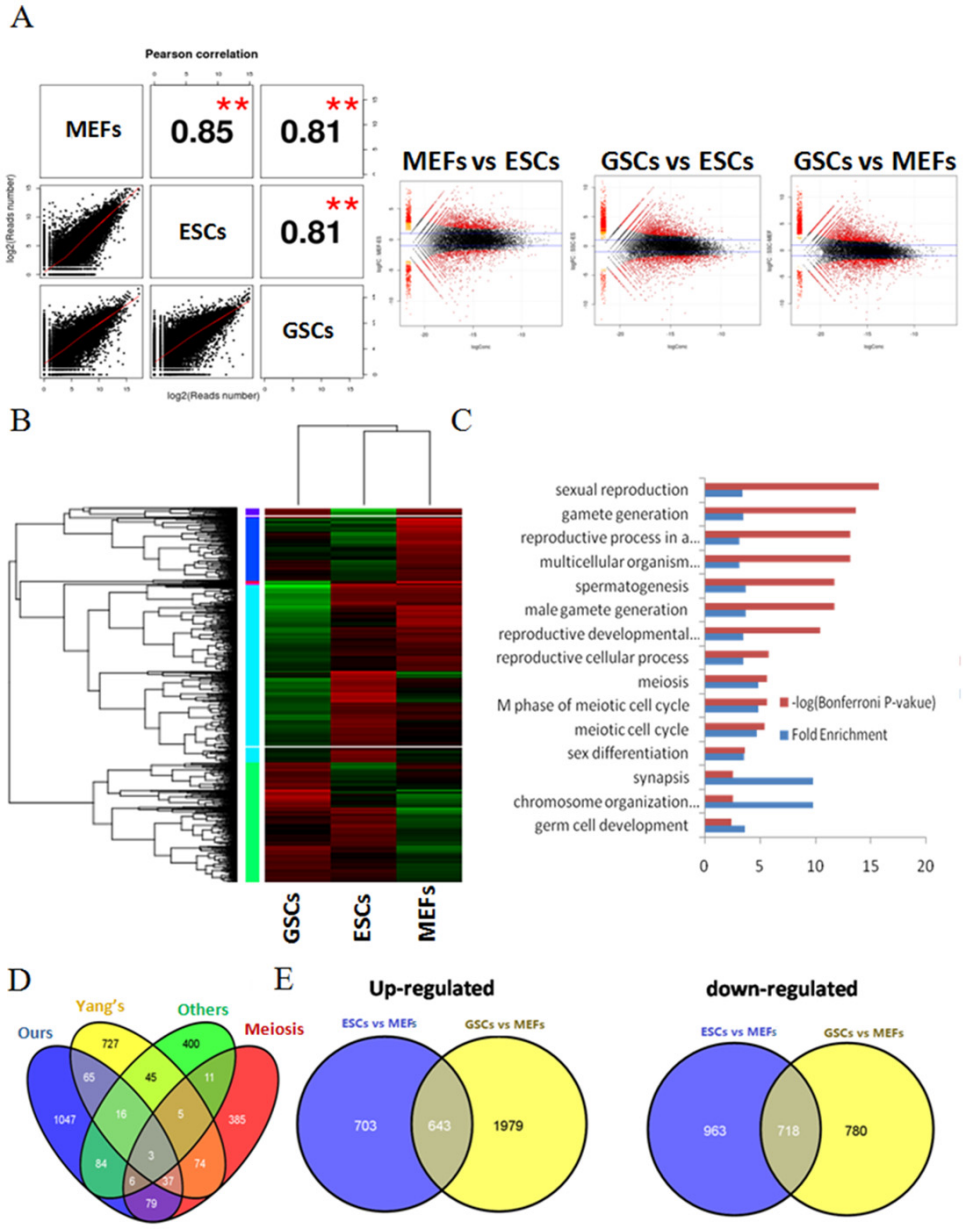
Differences in gene expression identified using SAPAS analysis. (A) Scatterplot and MA plot of gene expression using SAPAS in GSCs and ESCs. (B) Hierarchical clustering of differentially expressed genes among samples. (C) GO analysis of GSCs up-regulated (P<0.01, Fisher's exact test and fold change >2) in GSCs. A Bonferroni p-value < 0.001 corrects for errors due to multiple testing. (D) Venn diagram showing overlap between GSC up-regulated genes detected by SAPAS and other studies, including Yang’s [19], others [20–22] and meiosis [27]. (E) Venn diagram showing overlap between GSC and ESC up- or down-regulated genes compared to MEFs. GSCs, germline stem cells; MEFs, mouse embryonic fibroblast cells; ESCs, embryonic stem cells.

It has been reported that SAPAS and other 3'-end sequencing methods are as accurate as an RNA-Seq approach for digital gene expression. The normalized expression level of each gene was measured by reads per million mapped reads (RPM) in the gene. Considering that the reads counted from 3 samples were greater than 10, we detected 15,312–16,673 expressed genes in each sample, which included the majority of annotated mouse reference genes. The global profiles of gene expression were generally well correlated between samples, with Pearson correlation coefficients ranging from 0.81 to 0.85 (Fig 3A). We further analyzed the correlation with gene expression among different methods. The SAPAS data showed high agreement with RNA-Seq and array data in a ratio-based gene expression qualification comparison, and it was also well correlated with gene expression (Pearson r~0.7), indicating that our SAPAS data were accurate and efficient for quantitative analysis of gene expression.

Using the RPM expression data, differences in gene expression between GSCs and ESCs, ESCs and MEFs, and GSCs and MEFs were compared. We used edgeR [18] (FDR<0.01) to identify the differentially expressed genes (DEGs) between samples. Genes that changed more than 2-fold and had an FDR <0.01 were considered to be differentially regulated genes. The clustering analysis of DEGs indicates that the transcriptome of GSCs is distinguished from those of ESCs and MEFs (Fig 3B).

#### 1) Functional annotation of differentially expressed genes in GSCs vs. ESCs and MEFs

We identified 2301 genes that had higher expression and 1299 genes had lower expression in GSCs compared with ESCs (**Table D in** S1 File). Among differentially regulated genes, 1337genes were up-regulated and 443 genes were down-regulated in GSCs compared with ESCs and MEFs, and these are indicated as GSC-specific genes (**Table E in** S1 File). GSC-specific genes were evenly distributed among the chromosomes. To gain insight into the general functions of GSC-specific genes, we carried out GO functional annotation using DAVID bioinformatic resources. Consistent with the functions of the GSCs, the highly expressed genes in the GSCs were involved in not only the development of the spermatogonial system, indicated by terms such as "sexual reproduction", "spermatogenesis", and "gamete generation", but also several other processes related to various GSC functions, indicated by terms such as "meiosis" and “germ cell development" (Fig 3C, GO enriched terms are listed in Table 2). In contrast, the less-expressed genes in the GSCs were functionally different, i.e., mainly involved in cell adhesion and biological adhesion, which is in agreement with the nature of GSCs. We compared our data with a recent microarray dataset for GSCs. Of the 1133 GSC-specific genes, compared to differentiated cells, discovered by a previous study by Yang [19], 121 genes were also found to be GSC-specific genes in our SAPAS data. Our sequencing data had a wider dynamic range for gene expression and relative different ratio than the array data. Still, 109 out of the 570 genes previously characterized to be important for GSC maintenance were identified in our GSC-specific genes list [20–22]. The overlapping between the GSC-specific up-regulated genes in our study and other studies is shown as a Venn diagram (Fig 3D). Previous studies validate the significance of the findings in our study, and we newly detected many functionally related GSC genes.

**Table 2:** GO terms enriched and likely regulated by highly expressed GSC genes.

GO term	P Value	Fold Enrichment	Bonferroni*
sexual reproduction	1.49E-20	3.471985	3.80E-17
gamete generation	1.76E-18	3.542789	4.50E-15
reproductive process in a multicellular organism	6.56E-18	3.185719	1.67E-14
multicellular organism reproduction	6.56E-18	3.185719	1.67E-14
male gamete generation	6.89E-16	3.72274	1.70E-12
spermatogenesis	6.89E-16	3.72274	1.70E-12
reproductive developmental process	1.38E-14	3.525322	3.51E-11
reproductive cellular process	6.25E-10	3.55059	1.59E-06
meiosis	8.65E-10	4.864944	2.21E-06
M phase of meiotic cell cycle	8.65E-10	4.864944	2.21E-06
meiotic cell cycle	1.37E-09	4.756834	3.50E-06
sex differentiation	9.08E-08	3.579557	2.32E-04
synapsis	1.04E-06	9.854311	0.002655
chromosome organization involved in meiosis	1.04E-06	9.854311	0.002655
germ cell development	1.47E-06	3.685881	0.003741
extracellular matrix organization	6.16E-06	3.501587	0.01559

* Probabilities were evaluated by Bonferroni correction and values less than 0.01 were considered significant. GSCs, germline stem cells.

#### 2) Functional annotation analysis of differently expressed genes in GSCs and ESCs vs. MEFs

GSCs are adult stem cells that can both self-renew and differentiate into germ cells. Genes that are differentially regulated in both GSCs and ESCs may play an important role in pluripotency. By comparing the differentially expressed genes in GSCs vs. MEFs and in ESCs vs. MEFs, 643 genes were found to be highly expressed in both GSCs and ESCs, and 718 were down-regulated in these two cell lines (Fig 3E, **Table F in** S1 File). To better understand the function of the differentially regulated genes in both ESCs and GSCs, we conducted an enrichment GO analysis, as described above, for these genes. For the up-regulated genes, we observed many significant GO categories for GSC-specific genes, such as "sexual reproduction", "spermatogenesis", "male gamete generation", "reproductive cellular process" and "meiosis", suggesting that the up-regulation of GSC-specific genes is functionally related to GSC developmental processes (**Fig C in** S2 File). On the contrary, genes that were down-regulated in GSCs and ESCs were enriched in several functional processes related to tissue development and cell line differentiation (**Fig D in** S2 File).

### Verification of GSC specific genes that differentially expressed

**Fig 4:**
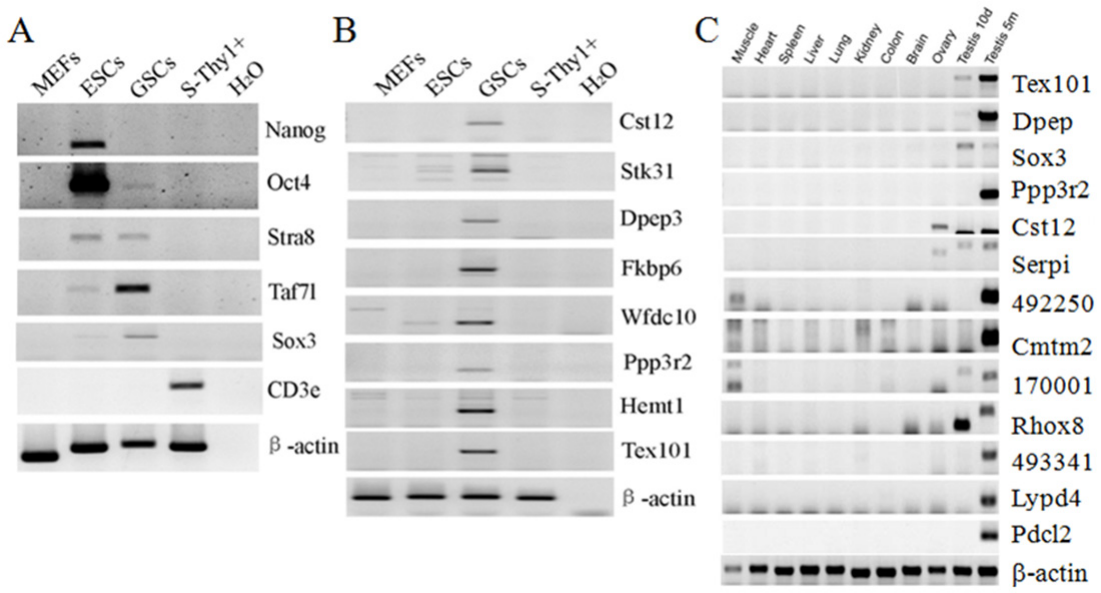
RT-PCR validation of specifically expressed GSC genes. (A) RT-PCR analysis of known GSC and ESC marker genes. Mouse spleens were digested and Thy1^+^ cells were isolated for the negative control (S-Thy1^+^). β-actin was used as an internal control. (B) RT-PCR validation of GSC specific genes by our SAPAS method. The 8 genes shown are from Table E in S1 File. β-actin was used as an internal control. (C) GSC-specific genes identified by our SAPAS methods were then RT-PCR detected indifferent mouse tissues, including young (10-day-old, 10d) and adult (5-month-old, 5m) testis.

To identify testis-specific genes in the mouse, we used experimental data based on EST data, microarrays and RNA-Seq profiles as gene expression data sources. Using the UniGene database for genes that contain ESTs, we identified 142 testis-specific genes [23]. We then collected a dataset of testis-specific genes from the TiSGeD database which mining of microarray datasets form more than 67 mouse tissues [24]. Still, testis-specific genes which were highly expressed in testis but lowly expressed in normal tissues or organs, were derived from data mining of 17 RNA-Seq dataset, which contain gene expression profiles of 6 different tissues (including brain, cerebellum, heart, kidney, liver and testis) [25]. Finally, we pooled data from these 3 datasets and compared GSC-up-regulated genes detected by our SAPAS data to identify GSC- and testis-specific genes. In all, 184 genes were found to match our expectations (**Table G in** S1 File). We performed RT-PCR to confirm the expression of 8 of these genes, in addition to the known markers Oct4, Stra8, Taf7l and Sox3 (Fig 4A and 4B). Because our GSCs were derived from 3-week-old mice, the GSCs were undergoing mitosis and meiosis by that timepoint, we also tested the expression of some genes in young (10-day-old) and adult (5-month-old) mice testis (Fig 4C). Most of the selected genes were expressed at higher levels in old mice testis, suggesting that they may be specific to mature or differentiating germ cells. GO analysis of those 184 overlapped genes showed that "sexual reproduction", "spermatogenesis", and "male gamete generation" were enriched, indicating that genes expressed at the highest levels in testis and GSCs are involved in spermatogenesis (**Fig E in** S2 File). Meiosis is a germ cell-specific cell division process and a necessary part of sexual reproduction [26]. Germ cells in the testis and ovaries undergo meiosis. A previous study identified 726 candidate meiosis-specific genes by using gene expression profiling of developing meiotic cells in the mouse [27]. By comparing our data to these meiosis-associated candidates, we found that 125 of them were up-regulated in our study (Fig 3D **and Table H in** S1 File). The GO categories of "meiosis", "sexual reproduction", and "male gamete generation"were enriched in these genes (**Fig F in** S2 File). In contrast, the 1070 remaining GSC up-regulated genes showed no overlap with meiosis-associated or testis-specific genes, but the GO categories of immune response, reproductive developmental process, sex differentiation, and extracellular structure organization were enriched among these GSC up-regulated genes (Benjamini <0.05) (**Table I in** S1 File, **Fig G in** S2 File).

### Verification of GSC specific genes that altered in alternative APA usage

**Fig 5:**
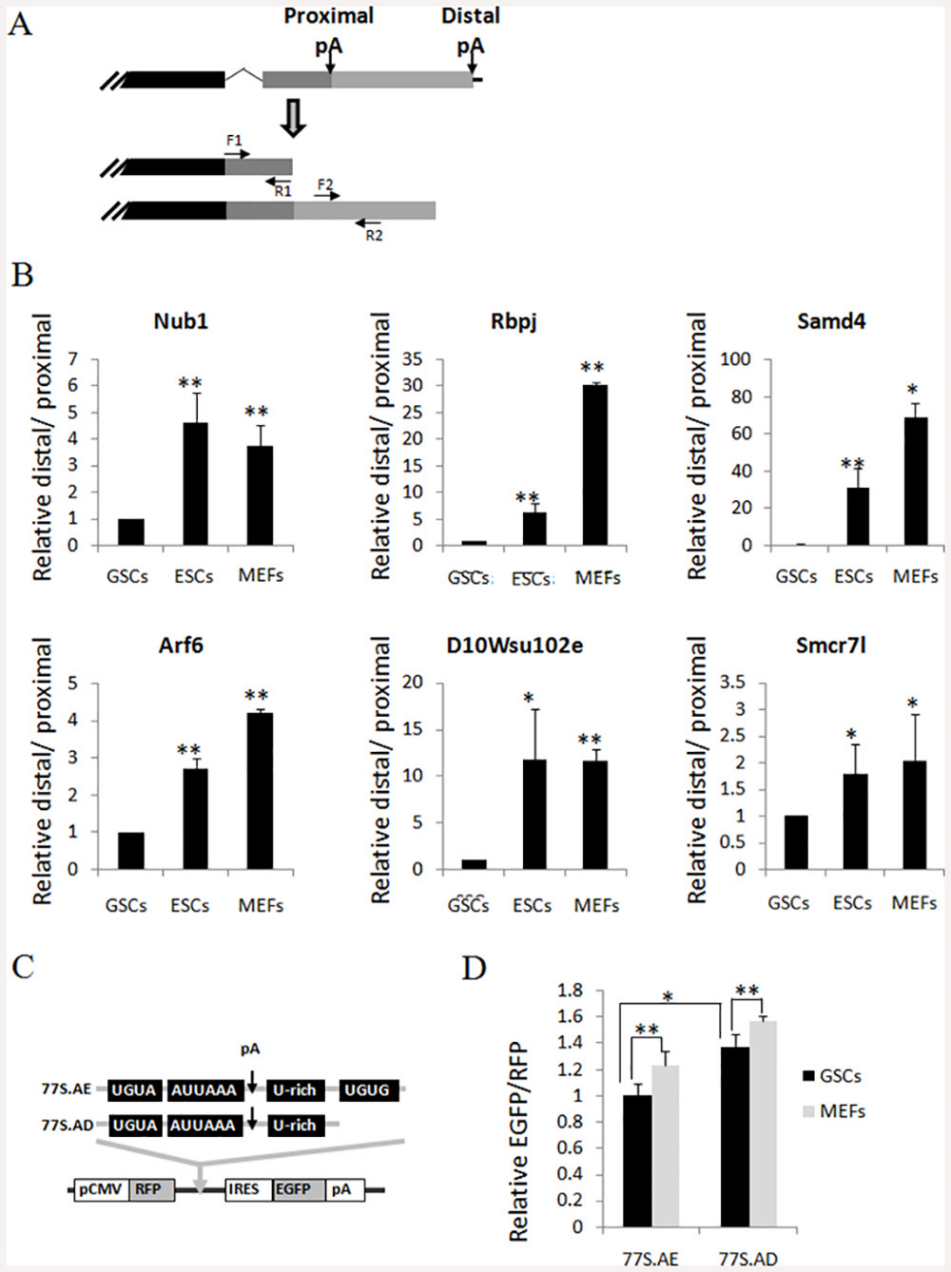
qPCR validation of altered APA events in GSCs. (A) Schematics of APA sites in the proximal and distal region of a gene. Two representative poly(A) sites are shown. F1/R1 Primers are designed within 1000bp upstream of the proximal sites while F2/R2 Primers are designed between two poly(A) sites (arrows). For each gene, Real-time PCR were performed using two pairs of primers F2R2 and F1R1, and the relative signal for primer set F2R2/F1R1 ratios was calculated. (B) qRT-PCR validation of 6 distal-to-proximal switch events in GSCs compared to ESCs and MEFs. Student’s T test, * P<0.05, ** P<0.01. (C) Schematics of APA reporter 77S.AD and 77S.AE [14]. (D) APA reporters were transfected into GSCs and MEFs. After 48 hours RNA was harvested and qRT-PCR was performed with primers targeting either the proximal (RFP) or distal (EGFP) regions of APA site, the relative expression levels of 2 transcript variants resulting from APA were calculated. Student’s T test, * P<0.05, ** P<0.01. GSCs, germline stem cells; MEFs, mouse embryonic fibroblast cells; ESCs, embryonic stem cells.

It was showed that compared to other tissues, the testis have the shortest 3'UTRs [14]. We found in this study that MEFs have the longest 3'UTR length in general (Fig 2A and 2B). Interestingly, compared to other two cells, GSCs has unique pattern in 3’ poly(A) usage. The 3'UTR shortened genes was more than the lengthened genes in GSCs vs. ESCs or GSCs vs. MEFs (Fig 2C). A total of 334 genes was found to switch to proximal pA sites (**Tables H and I in** S1 File). qRT-PCR test confirmed six of these genes were specifically shortened in GSCs compared to ESCs and MEFs (Fig 5A and 5B), suggesting our analysis methods work well in detecting APA switch events as well as profiling the expression level.

To confirm the regulation of APA in GSCs, we also used reporter constructs, which can generate 2 transcript variants by using either proximal or distal poly(A) site (Fig 5C) [14]. As previously 77S.AD had lower polyadenylation activity than 77S.AE in both cells [14]. Significantly, polyadenylation took place more frequently at the proximal poly(A) site in GSCs than in MEFs (Fig 5D), suggesting GSCs had relative shorter 3'UTR than MEFs. These results verified our SAPAS data in experiment. Moreover, we performed the cis elements analysis for regions surrounding the proximal poly(A) site used preferentially in GSCs compared to ESCs and MEFs. Using Multiple Em for Motif Elicitation (MEME) Software, our results show that C-rich elements were enriched (**Fig H in** S2 File). The function of C-rich elements were unknown yet and our results indicate C-rich elements may regulate polyadenylation in GSCs.

## Discussion

In this study, we used a high-throughput method, SAPAS, to detect poly(A) sites and to quantify APA isoform expression in GSCs, ESCs and MEFs. We found that GSCs tend to have shorter 3'UTR lengths, while MEFs tended towards longer 3'UTR lengths.

During mouse embryonic development, a zygote forms by the fusion of male and female germ cells that originate from GSCs in each sex. Three days later, the combined cells develop into a blastocyst, from which ESCs are derived. MEFs were derived from E12.5 embryos. The 3'UTR length in the three cell types identified in this study seems to be negatively correlated with their developmental stage. Previously, researchers found that states including increased proliferation, de-differentiation, and diseases such as cancer are associated with general 3'UTR shortening, while late developmental stage correlates with longer 3'UTRs [8,17]. During iPS reprogramming of cells from the testis, 3'UTR lengthening was also observed, suggesting that isolates from the testis tend to have the shortest 3'UTRs [14]. During spermatogenesis, in the transition from spermatogonia to mature spermatids, the 3'UTR tends to become shorter, and this partially coincides with protein level changes of mRNA processing factors, according to a systematic analysis of APA site usage in mouse testicular cells at various stages of spermatogenesis (spermatogonia, spermatocytes and round spermatids) [28].

Polyadenylation is a transcription-coupled event that ensures that mRNAs remain stable and intact. Alternative polyadenylation yields a dynamic 3'UTR length, which could affect mRNA stability, mRNA transport, or translation initiation. Alternative polyadenylation regulates gene expression in many tissues, including male germ cells, to control the specificity of protein expression [29]. It has been shown that mRNA with a shorter 3'UTR produces more proteins. In male germ cells, the mRNA tends to undergo APA more often than in cells in other tissues [30], but the polyadenylation signal AAUAAA is less used [28, 31]. The detailed mechanisms, functions and consequences of APA regulation in germ cells remains unknown, but evidence is emerging showing that APA switching in male germ cells may regulate male reproductive ability. For example, the loss of testis-specific Cstf64 in mice caused male infertility resulting from spermatogenesis defects. Extensive analysis revealed that this may have been due to the deficient 3' processing of some directly regulated genes or from a distal switch at APA sites of other genes [32, 33]. Similarly to what has been observed in germ cells, mRNAs in tumor cells also have relatively shorter 3'UTR lengths compared to normal cells. A shorter 3'UTR may simplify the post-transcriptional regulation of GSC-specific genes, allowing the cells to precisely and quickly turn on/off the related genes during germ cell development. However, an on/off gene control mechanism does not fully explain the complex process of tumor formation. In tumor cells, there is much more complicated and comprehensive variation in gene isoforms, and tumors develop many more signaling pathways and dynamic DNA/RNA/protein networks to fit their flexible transformation. Many proto-oncogenes drive tumor transformation by altering mRNA variation to a shorter 3'UTR [8]. The idea of APA regulation may be striking the collective imagination in that it was recently found that knocking down CFIm25, a repressor of proximal APA sites, could enhance tumorigenesis whereas its overexpression reduced tumor properties [34].

In addition, we also identified GSC-specific genes. Of these, 1337 genes were up-regulated and 443 genes were down-regulated in GSCs compared with MEFs and ESCs. These genes partially overlapped with those identified in previous reports (Fig 3D), suggesting that many potentially novel genes were identified by our research. Moreover, our work identified many GSC-specific genes which use shorter 3’UTR than ESCs or MEFs and they could be confirmed by qRT-PCR validation as well as APA reporter assay. Also the cis element analysis indicated that C-rich elements may specifically regulate polyadenylation in GSCs. So far, several proteins such as Pcbp2, Tfcp2 and Hnrnp-E1 were found to associate with C-rich elements around poly(A) site and they may function in control of 3' processing, RNA stability and translation [35]. Further studies using genetically knockout (KO) tissues for SSC transplantation, KO mouse phenotypic analysis and methods such as clustered regularly interspaced short palindromic repeat (CRISPR)/Cas9 mediated gene editing, systematic evolution of ligands by exponential enrichment (SELEX) as well as UV cross-linking and immunoprecipitation (CLIP) could identify the functions and mechanisms of trans elements and cis elements in the near future. The advancement of high-throughput sequencing techniques has also dramatically improved biomedical research in recent years, especially in studies of gene expression profiles. Recently, stage-specific profiles of GSCs and spermatogenesis process in DNA methylation, 5hmC, histone modifications/variants and RNA-seq were performed [36]. Our work focused on the gene expression and APA isoform identification. More profiling methods, such as crosslinking immunoprecipitation of RNA binding proteins coupled with high-throughput sequencing, will also be of benefit for identifying the cis- and trans-regulatory mechanisms involved in APA. It is worth mentioning that APA regulation study is not restricted to coding mRNAs. More investigation of the APA site-switching in the lncRNAs we identified could shed light on the roles of lncRNA genes with differing 3'UTR lengths at distinct developmental stages.

## Supporting Information

S1 File Table A, Top detected PAS hexamers. Table B, 334 genes list of GSCs-specific genes with shortened 3'UTR usage. Table C, 168 genes list of GSCs-specific genes with lengthened 3'UTR usage. Table D, Differentially expressed genes in GSCs compared to ESCs. Table E, List of GSC up-and down-regulated genes (1337 up and 443 down). Table F, GSC and ESC both up- and down-regulated genes compared to MEFs. Table G, List of 184 Genes that are both GSC up-regulated and testis up-regulated. Table H, List of 125 genes that are both GSCs up-regulated and meiosis-associated. Table I, List of 1102 genes that are GSC up-regulated and do not overlap with meiosis-associated and testis-upregulated genes.
(XLSX)

S2 File Fig A, GO analysis of 334 genes with up-regulated proximal poly(A) in GSCs. Fig B, GO analysis of 239 genes with poly(A) distal-to-proximal switch events that happened in both GSCs vs. MEFs and ESCs vs. MEFs comparisons. Fig C, GO analysis of 643 GSCs and ESCs up-regulated genes compared to MEFs. Fig D, GO analysis of 718 GSCs and ESCs down-regulated genes compared to MEFs. Fig E, GO analysis of 184 up-regulated GSC- and testis-specific genes. Fig F, GO analysis of 125 up-regulated meiosis-specific genes in GSCs. Fig G, GO analysis of 1070 up-regulated non meiosis-specific genes in GSCs. Fig H, Cis elements analysis for five regions surrounding the poly(A) site used preferentially in GSCs compared to ESCs and MEFs.
(PDF)
