# Supplementary material for: Specific Tandem 3'UTR Patterns and Gene Expression Profiles in Mouse Thy1+ Germline Stem Cells
Source: PLoS One. 2015 Dec 29;10(12):e0145417. doi: 10.1371/journal.pone.0145417 (PMC4699828; doi:10.1371/journal.pone.0145417)
Supplement: S2 File — Fig A, GO analysis of 334 genes with up-regulated proximal poly(A) in GSCs. Fig B, GO analysis of 239 genes with poly(A) distal-to-proximal switch events that happened in both GSCs vs. MEFs and ESCs vs. MEFs comparisons. Fig C, GO analysis of 643 GSCs and ESCs up-regulated genes compared to MEFs. Fig D, GO analysis of 718 GSCs and ESCs down-regulated genes compared to MEFs. Fig E, GO analysis of 184 up-regulated GSC- and testis-specific genes. Fig F, GO analysis of 125 up-regulated meiosis-specific genes in GSCs. Fig G, GO analysis of 1070 up-regulated non meiosis-specific genes in GSCs. Fig H, Cis elements analysis for five regions surrounding the poly(A) site used preferentially in GSCs compared to ESCs and MEFs. (PDF) [file pone.0145417.s002.pdf]

Figure A

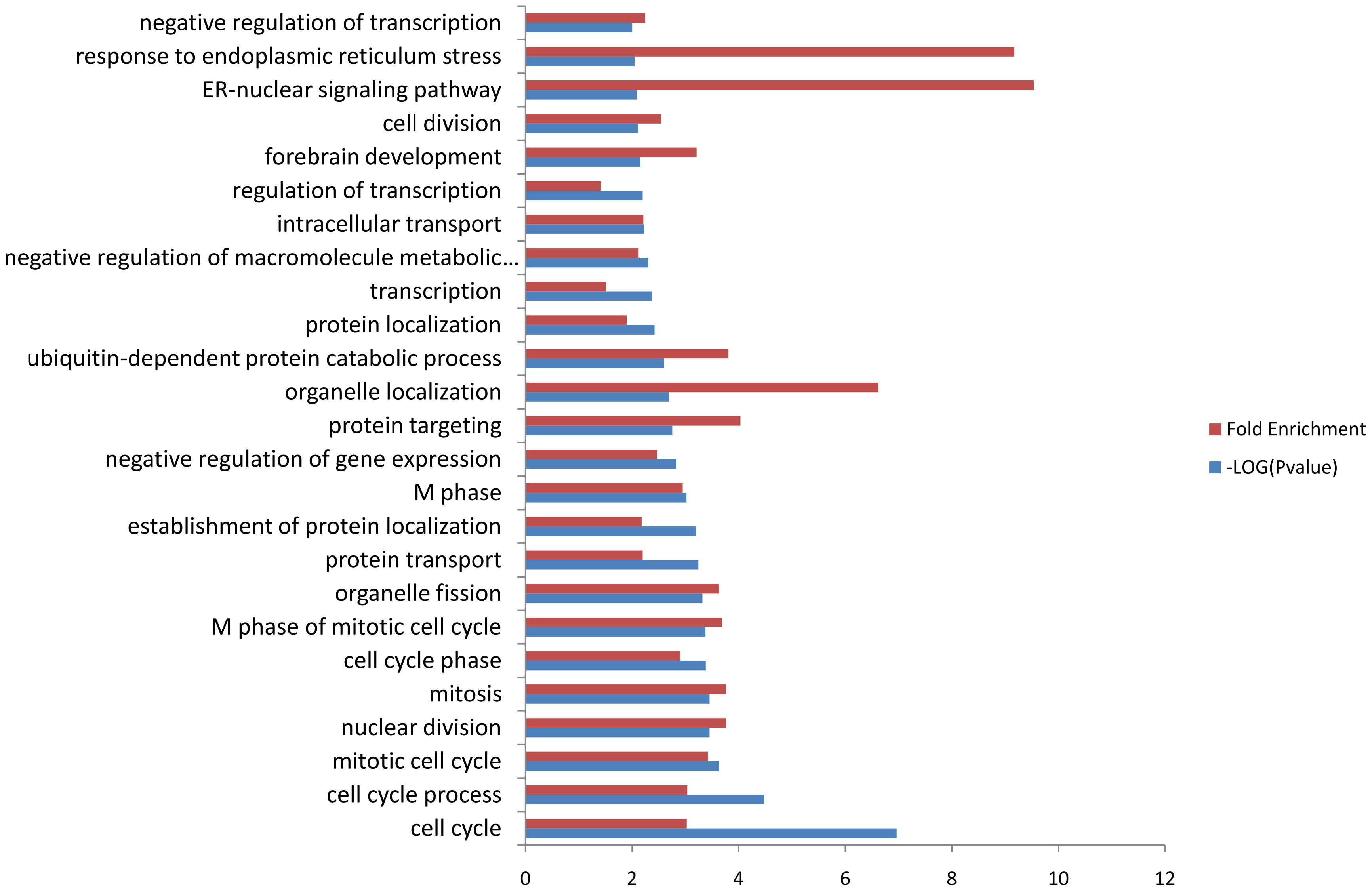

Figure A in S2 File. GO analysis of 334 genes with upregulated proximal pA in GSCs.

Figure B

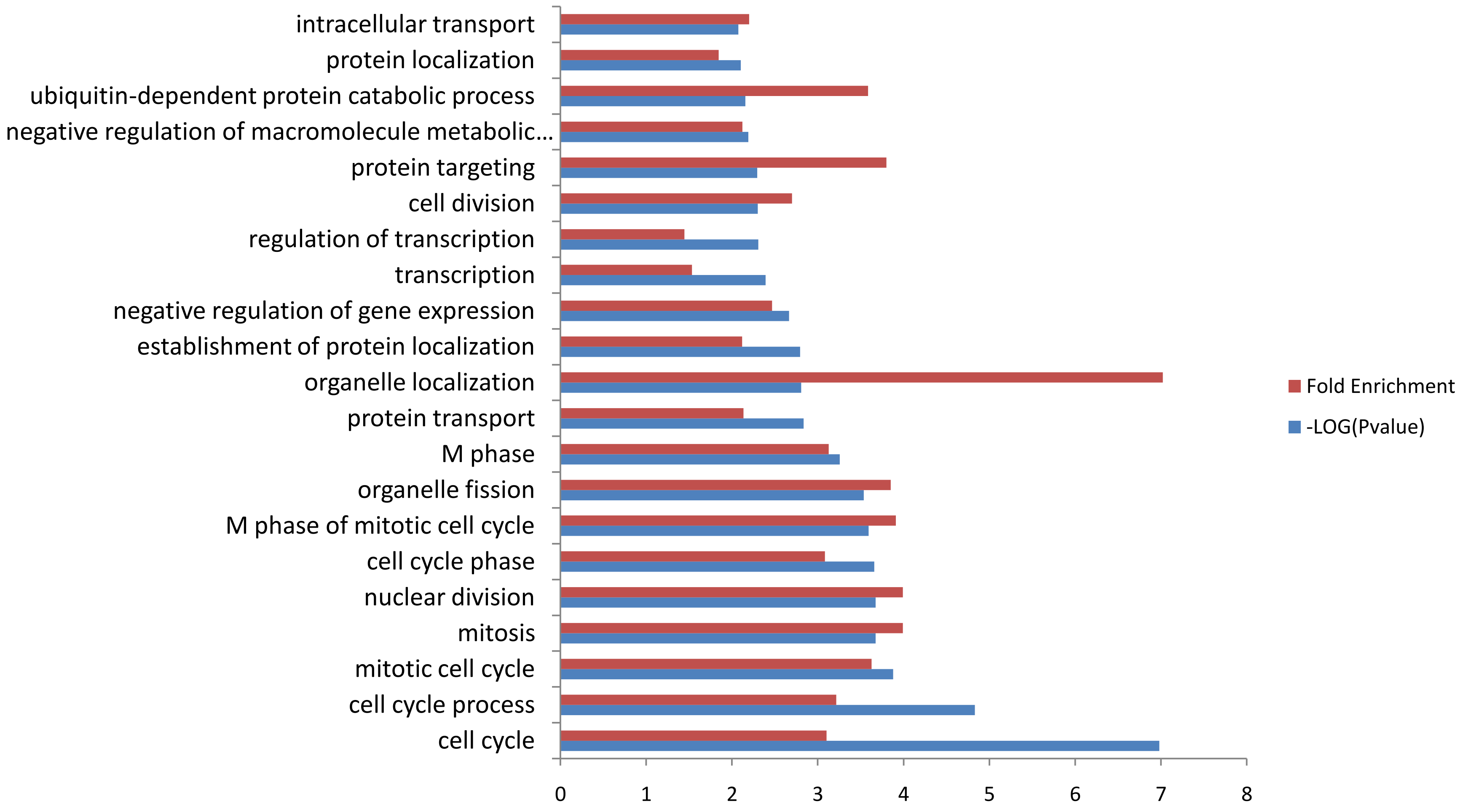

Figure B in S2 File. GO analysis of 239 genes with 3'UTR distal-to-proximal switch events happened both in GSCs vs MEFs and ESCs vs MEFs comparisons.

Figure C

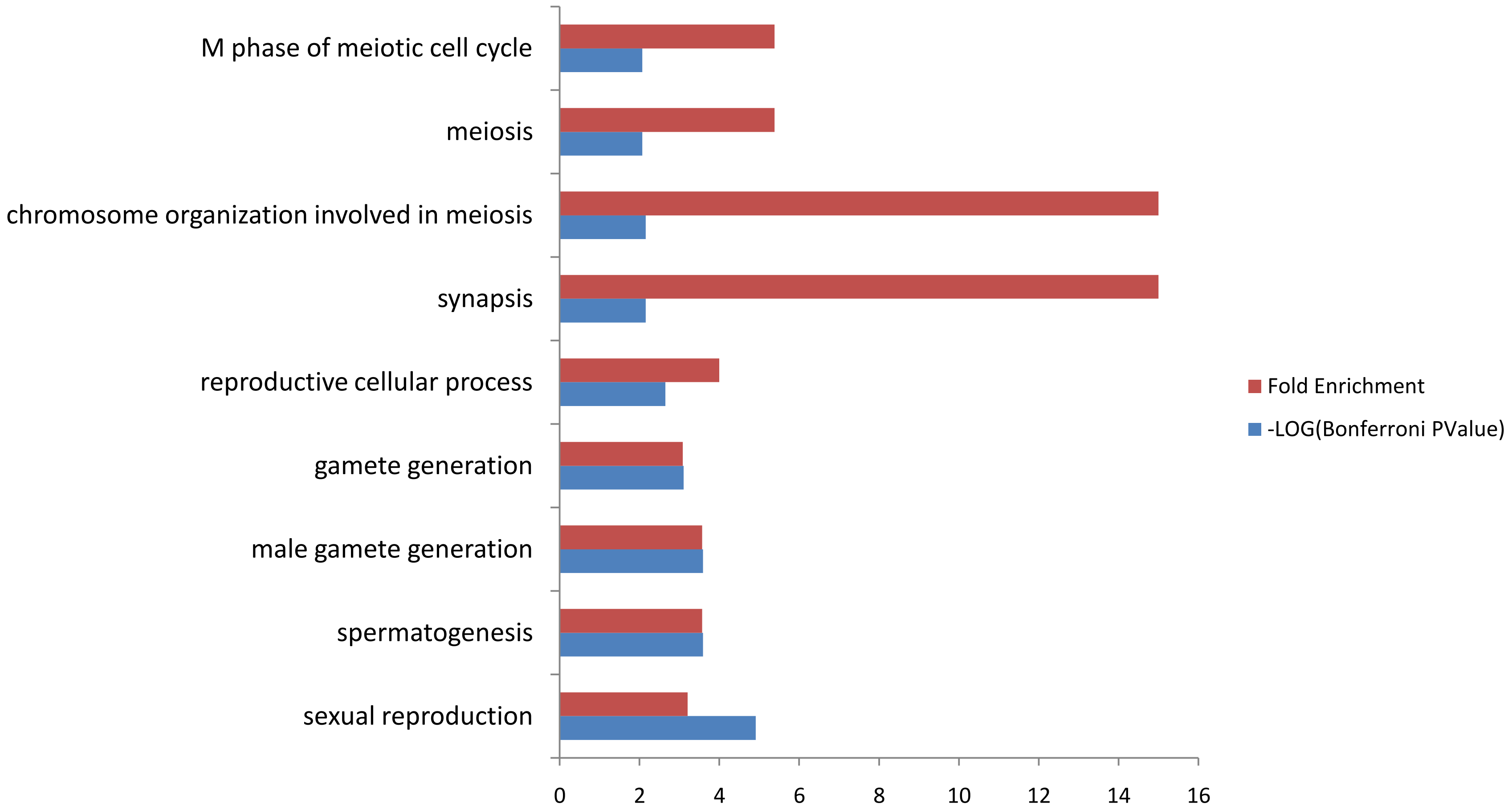

Figure C in S2 File. GO analysis of 643 GSCs and ES up-regulated genes as compare to MEFs.

Figure D

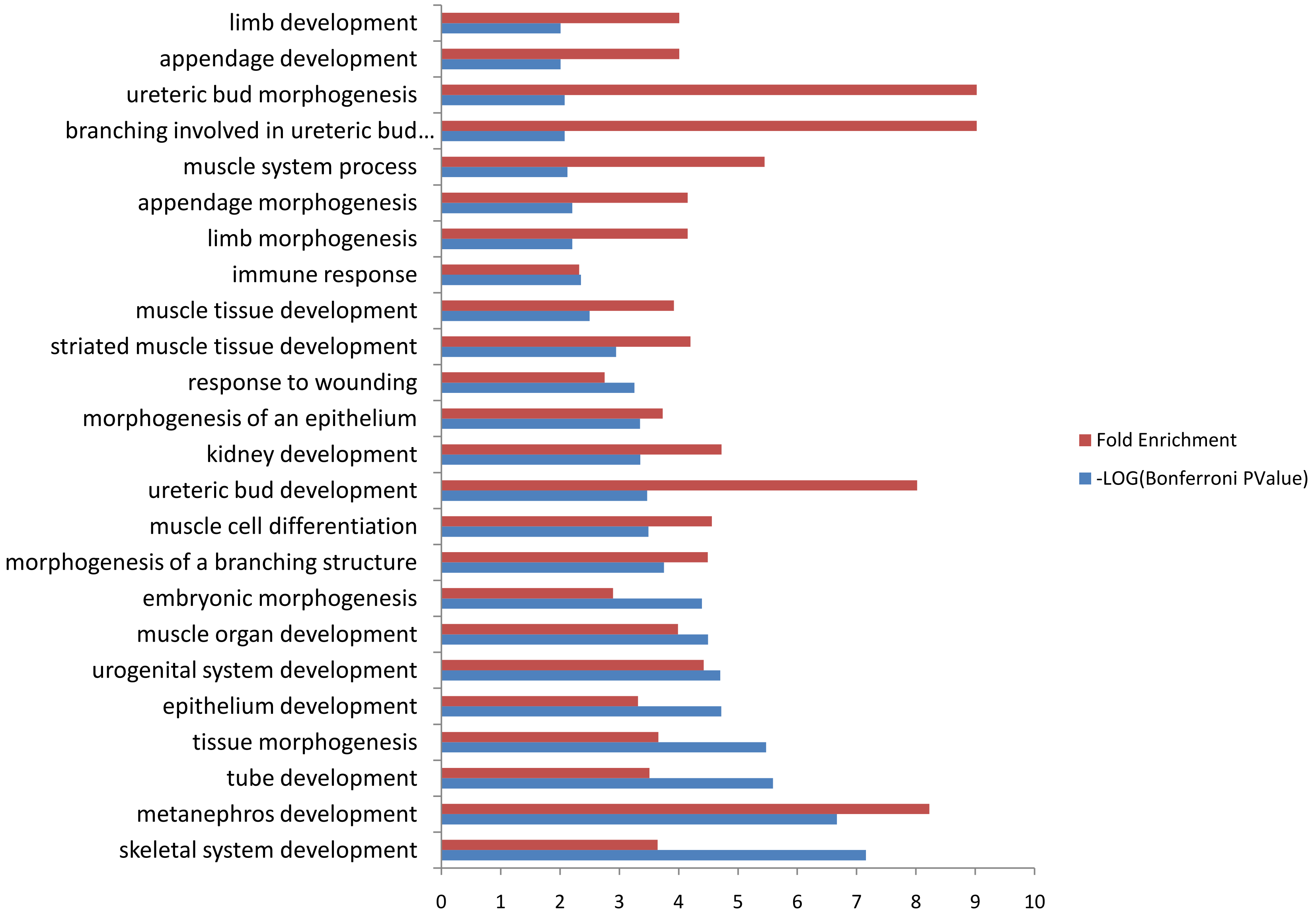

Figure D in S2 File. GO analysis of 718 GSCs and ES down-regulated genes as compare to MEFs.

Figure E

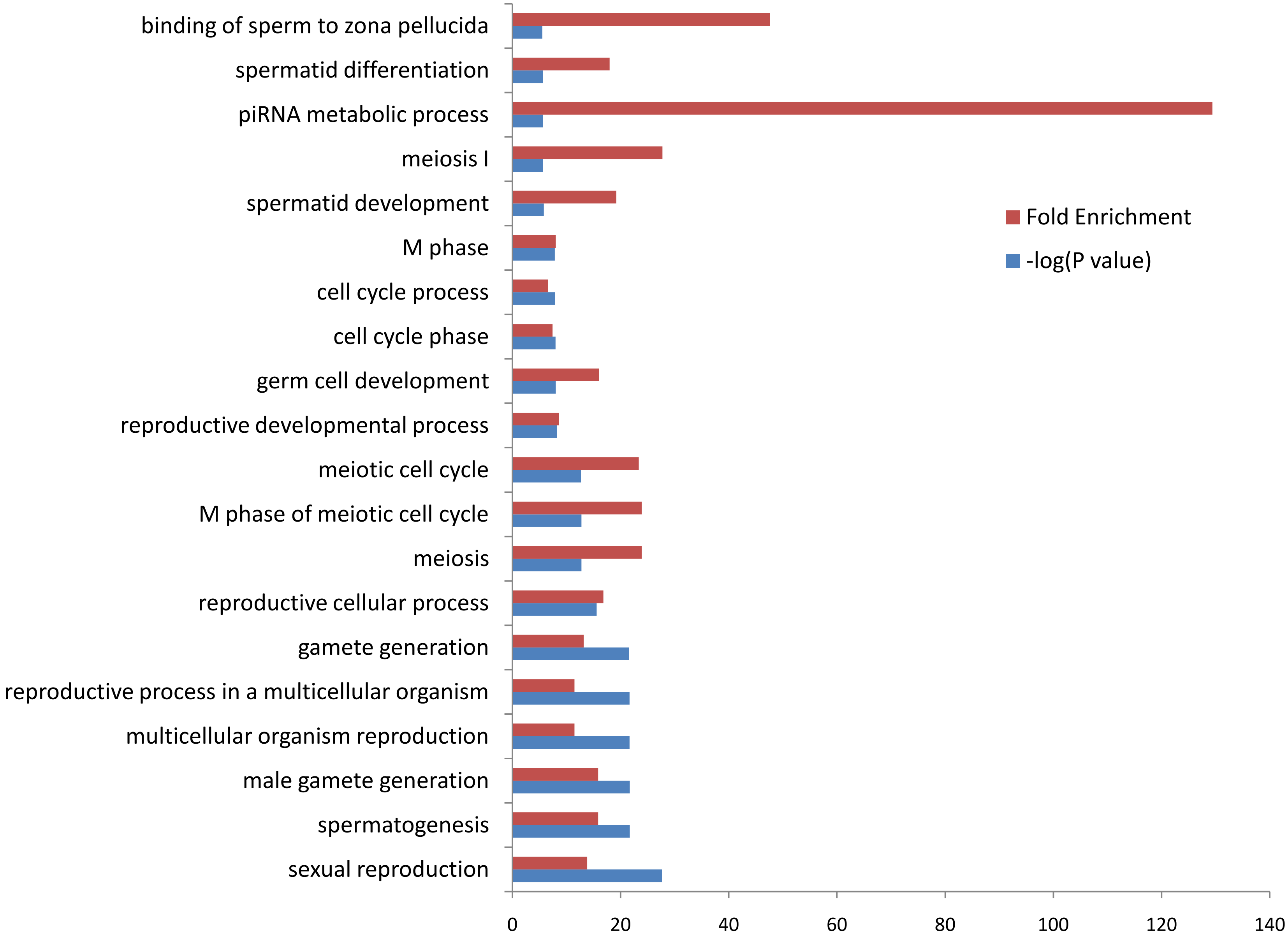

Figure E in S2 File. GO analysis of 184 up-regulated GSC- and testis-specific genes.

Figure F

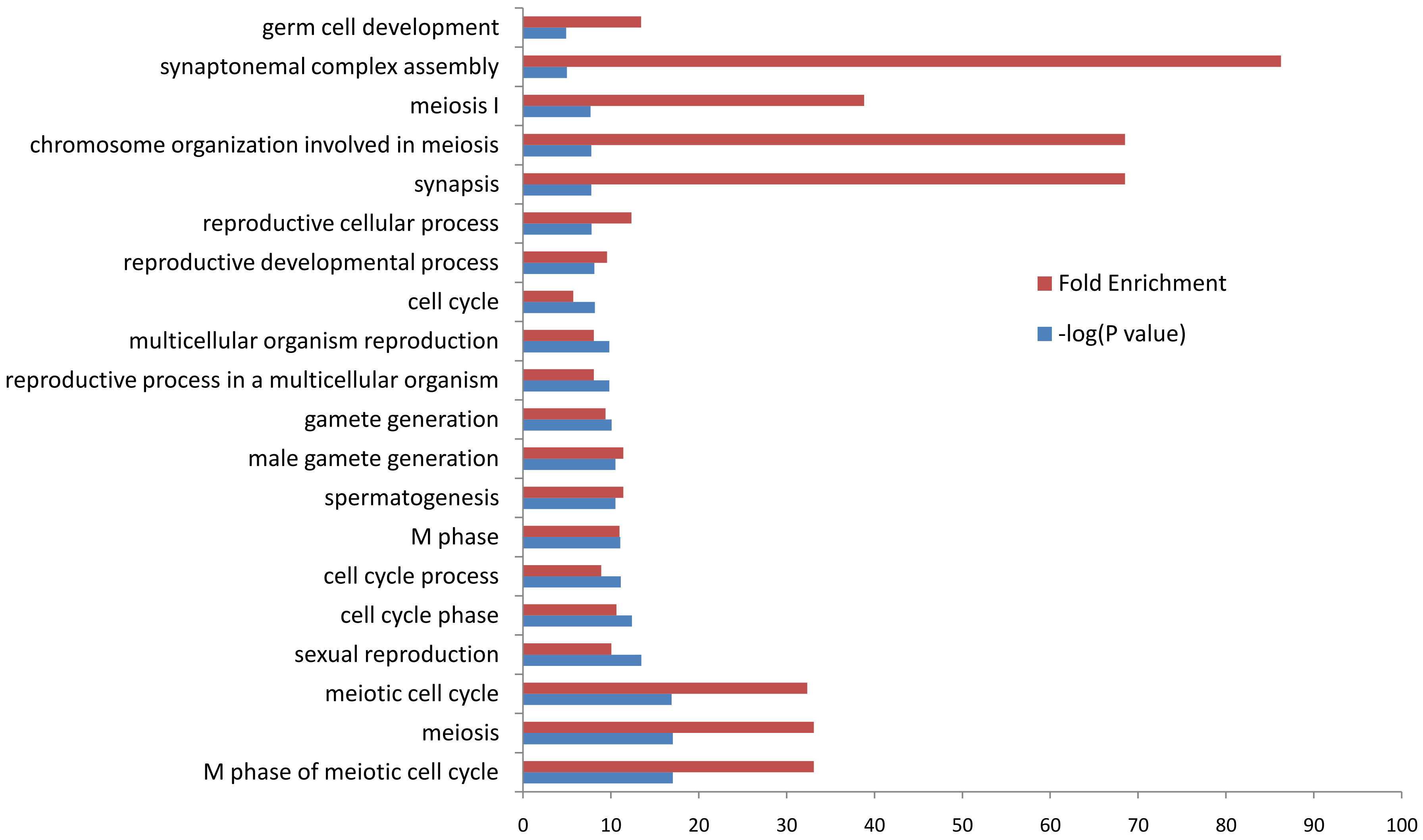

Figure F in S2 File. GO analysis of 125 up-regulated meiosis-specific genes in GSCs.

Figure G

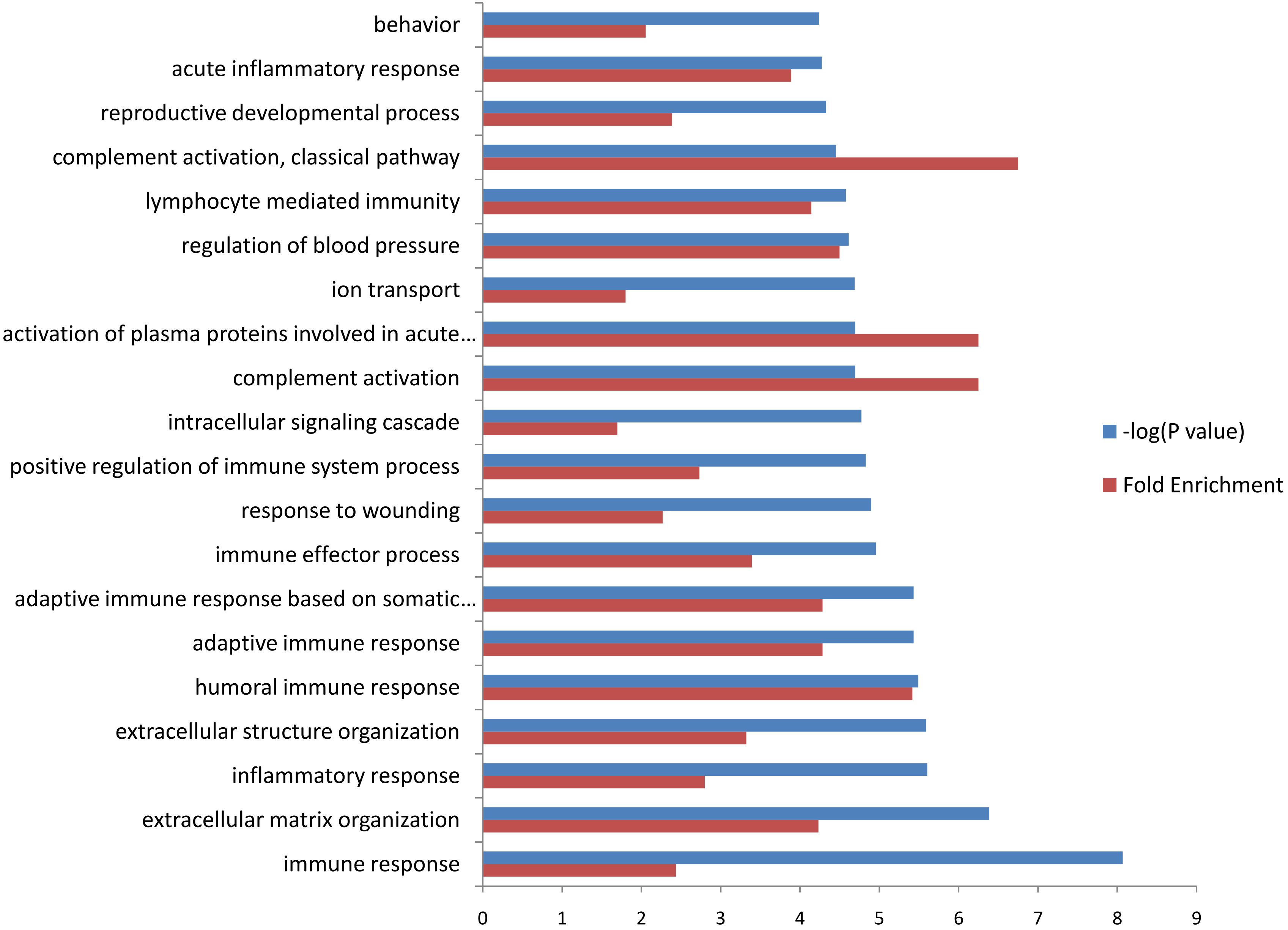

Figure G in S2 File. GO analysis of 1070 up-regulated non meiosis-specific genes in GSCs.

Figure H

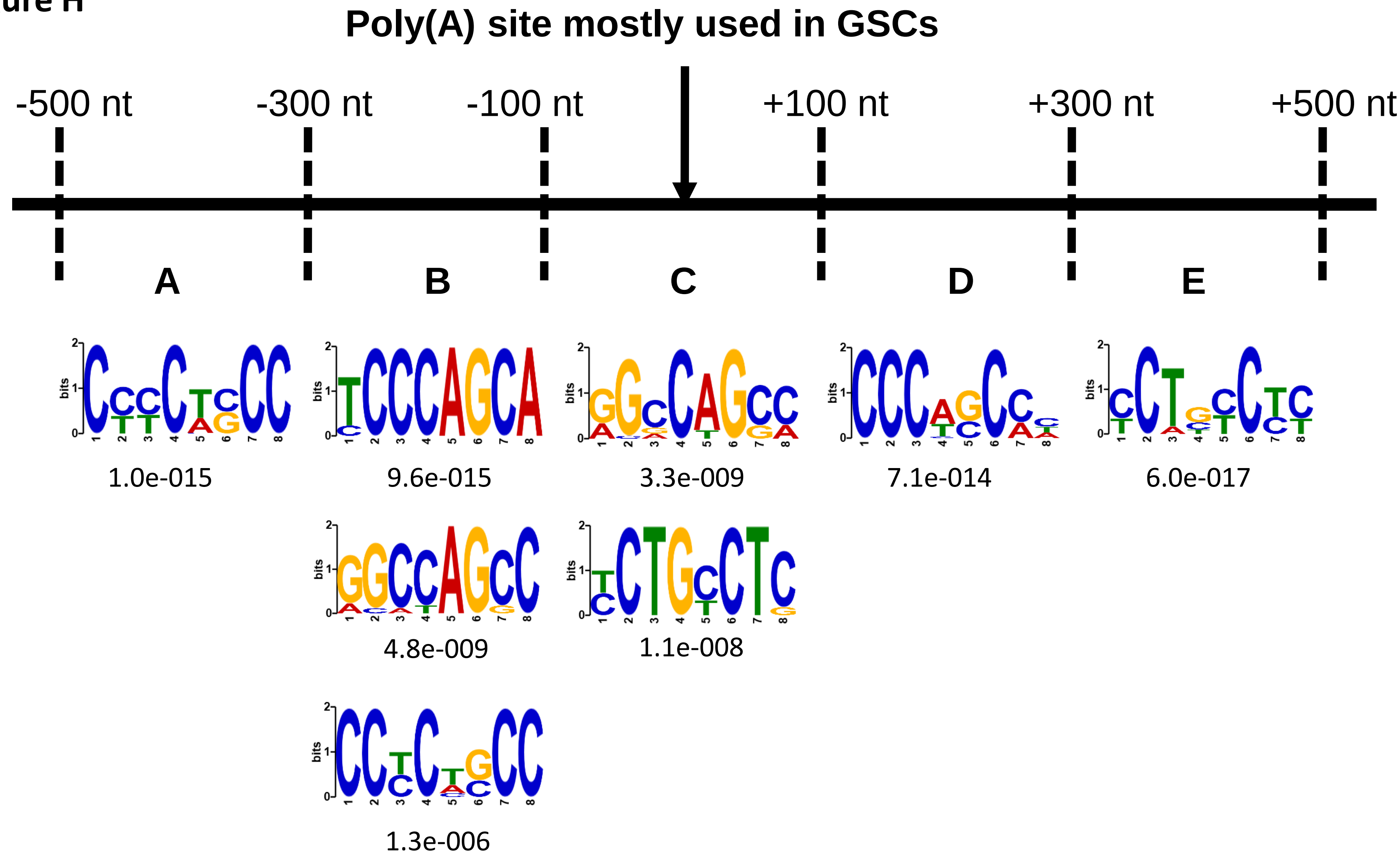

Figure H in S2 File. Cis elements analysis for APA. Five regions surrounding the poly(A) site used preferentially in GSCs compared to ESCs and MEFs were shown. Five subregions A,A,C,D,E were devided in the -500 and +500 to the APA site. Motifs were analyzed using Multiple Em for Motif Elicitation (MEME) Software. E values representing the statistical significance of the motif were shown below. The most statistically significant (low E-value) motifs were shown first.
